# Prosociality differences in neural reward processing during cooperation and competition: an ERP investigation

**DOI:** 10.3389/fpsyg.2026.1982610

**Published:** 2026-10-29

**Authors:** Jiachen Lu, Yiwen Li, Jinting Qiu, Chunyu Li

**Affiliations:** School of Education, Guangzhou University, Guangzhou, China

**Keywords:** competition, cooperation, event-related potential, prosociality, reward processing

## Abstract

**Introduction:**

Reward evaluation is shaped by social context and individual traits, but how prosociality modulates neural gain–loss differentiation during real interpersonal cooperation and competition remains unclear. This study examined the feedback-related negativity difference wave (dFRN) in cooperative and competitive tasks.

**Methods:**

Forty participants (20 females; mean age = 22 years) completed face-to-face cooperation and competition tasks while EEG was recorded. Prosociality was measured with the Prosocial Scale. dFRN was calculated as loss-evoked minus gain-evoked mean voltage at Fz, FCz, and Cz within 220–300 ms after reward feedback. A 2 (Social Context) × 2 (Prosociality) mixed ANOVA and linear mixed-effects models using continuous prosociality scores were conducted.

**Results:**

A significant Social Context × Prosocial Trait interaction emerged. High-prosocial participants showed a more negative dFRN in cooperation than in competition, whereas low-prosocial participants showed no reliable context difference. Continuous-score mixed-effects models adjusted for sex supported this association (b = −0.315 μV per scale point, SE = 0.152, z = −2.07, *p* = 0.039, 95% CI [−0.614, −0.016]); the group-based contrast (*p* = 0.054) and robust-standard-error analysis (*p* = 0.051) were marginal.

**Discussion:**

These findings suggest that prosociality is associated with context-dependent reward differentiation, with shared cooperative outcomes eliciting stronger loss–gain differentiation among high-prosocial individuals. Causal interpretation is limited by the fixed task order, differing outcome-generation rules, and unmodeled dyadic dependence.

## Introduction

Reward evaluation is a core cognitive process through which individuals utilize reward information to guide goal-oriented behaviors (Ressler, 2004; Ballard et al., 2024). In the complex social life of humans, there are diverse ways to obtain rewards, which can be achieved through either cooperation or competition. Therefore, reward evaluation does not occur in isolation but is significantly influenced by the environment in which rewards are obtained (Takagaki and Krug, 2020). This view has been supported by numerous cognitive neuroscience studies (Kujawa, 2024). Moreover, research has shown that compared with the cooperative environment, the competitive environment enhances individuals’ sensitivity to rewards (Czeszumski et al., 2019).

Additionally, prosocial traits may influence reward evaluation (Hui et al., 2020). Prosociality encompasses behaviors motivated by concern for others’ welfare (Feigin et al., 2014; Thielmann et al., 2020; Rodrigues and Hewig, 2021). High-prosocial individuals tend to consider both self- and other-related consequences when evaluating rewards (Li et al., 2020; Lu et al., 2022). Cooperative environments that align with this orientation may therefore enhance reward sensitivity, whereas competitive contexts that conflict with it may attenuate sensitivity. This suggests that prosocial traits and social context may jointly shape reward evaluation.

In the field of cognitive neuroscience, the neural mechanisms of reward evaluation have been extensively studied. Functional magnetic resonance imaging (fMRI) studies have shown that reward evaluation involves multiple brain regions, including the anterior cingulate cortex (ACC), the orbitofrontal cortex (OFC), and the striatum (Haber and Knutson, 2010). Event-related potential (ERP) studies have found that the feedback negativity (FRN) is closely related to reward evaluation, and the loss of a reward usually induces a strong FRN amplitude (Lei et al., 2019). The FRN is a negative ERP component that peaks between 200 and 350 milliseconds after the presentation of feedback stimuli, and its maximum amplitude usually occurs in the mid-frontal region (Sambrook and Goslin, 2015; Baker and Holroyd, 2011; Rodrigues et al., 2024). Notably, the FRN has been referred to by various names (Krigolson, 2018), such as feedback negativity (FN: e.g., Bress et al., 2015), medial frontal negativity (MFN: e.g., Gehring and Willoughby, 2002; Boksem and De Cremer, 2010, Wang et al., 2016), and feedback error-related negativity (fERN: e.g., Holroyd and Coles, 2002); additionally, based on accumulating evidence, there is a growing push to term the FRN as reward positivity (RewP, Baker and Holroyd, 2011; Proudfit, 2015) or correct-related positivity/feedback correct-related positivity (Holroyd et al., 2008). For the present study, the key issue is how strongly neural responses distinguish reward losses from gains.

This distinction is captured by the FRN difference wave (dFRN), which contrasts loss- and gain-related activity (Holroyd et al., 2008). When calculated as loss minus gain, a more negative dFRN indicates stronger neural differentiation between these outcomes. It may reflect a more negative response to losses, a more positive response to gains, or both (Proudfit, 2015). Such differentiation may depend on how outcomes are anticipated and valued: FRN responses are sensitive to reward magnitude and probability (Sambrook and Goslin, 2015), and reduced feedback negativity has been associated with lower subjective involvement (Yeung et al., 2005). These findings suggest that expectations and motivational significance may shape the loss–gain contrast (Oliveira et al., 2007), although dFRN alone cannot distinguish their contributions. Because our research question concerns whether prosociality alters the differentiation of gains and losses across cooperation and competition, we use dFRN as the principal ERP outcome measure.

As an external stimulus, the meaning and impact of rewards vary depending on the context. Reward evaluation is not only influenced by the valence of rewards but also regulated by environmental factors and individual traits. For example, Kimura and Katayama (2016) found through a gambling task that in a cooperative environment, when participants shared monetary outcomes with group members, the FRN amplitude related to monetary outcomes was significantly reduced, while in an individual environment, the FRN amplitude did not change significantly. The study by Lei et al. (2019) further showed that the cooperative environment can partially regulate the FRN amplitude induced by the loss of rewards. The authors explained that the cooperative relationship may reduce the sensitivity to reward gains and losses. In addition, the competitive environment has been found to enhance individuals’ neural responses (Lin and Liang, 2024). For instance, the study by Van Meel and Van Heijningen (2010) showed that compared with performing tasks independently, individuals had a stronger FRN amplitude induced by the loss of rewards in a competitive environment. The study by Czeszumski et al. (2019) also supported this finding. They compared the FRN amplitudes of 26 pairs of participants in competitive and cooperative situations, and the results showed that the FRN amplitude in competitive trials was significantly higher than that in cooperative trials.

Prosociality, as an individual trait, has become a hot topic in reward evaluation research in recent years. For example, Gan et al. (2016) used ERP technology to study the neural correlates of the evaluation of the outcomes of helping others in a decision-making task and found that the FRN amplitude induced by failure outcomes (indicating failure to help others) was significantly larger than that induced by success outcomes (indicating success in helping others). Qi et al. (2018) found in a social comparison task that only high-prosocial individuals could still pay attention to others’ gains and losses when they themselves lost rewards. Lu et al. (2022) found through a face-to-face point-comparison task that high- and low-prosocial individuals differed in their FRN responses to card outcomes during competition. These findings concern card-outcome feedback preceding the final reward feedback, rather than a direct comparison of cooperative and competitive reward responses.

In summary, existing studies have shown that when evaluating reward outcomes, individuals not only focus on the valence of rewards but are also significantly influenced by environmental factors. For example, the cooperative environment may reduce individuals’ sense of loss when losing rewards due to the dispersion of responsibility or peer relationships, while the competitive environment may increase individuals’ sensitivity to rewards by enhancing the level of motivation. Notably, prosocial traits may play a key regulatory role in this process: high-prosocial individuals show enhanced attention to collective rewards in cooperative tasks and may exhibit adaptive adjustments in motivation levels due to value conflicts in competitive tasks. However, previous studies have either examined the cooperative situation (Lei et al., 2019) or the competitive situation (Lu et al., 2022) in isolation, or compared reward processing differences between the two situations (Czeszumski et al., 2019) without exploring individuals’ prosocial traits; additionally, in prior research, participants typically completed tasks independently or interacted with computers to control variables (Wang et al., 2017; Liu et al., 2021; Tor and Garcia, 2023), despite evidence showing that humans exhibit significantly different behavioral patterns and brain responses when interacting with real people compared to when interacting with computers (Cui et al., 2012; Rilling and Sanfey, 2011).

In view of this, the present study adopted a real interpersonal interaction paradigm involving cooperation and competition, combined with high-temporal-resolution ERP technology. In the cooperation task, both participants selected cards simultaneously and received the same feedback determined by their joint total, creating outcome-level interdependence without requiring strategic negotiation. Social value orientation theory suggests that prosocial individuals value joint outcomes (Van Lange, 1999; Balliet et al., 2009; Hilbig et al., 2014). For these individuals, shared gains and losses may reinforce the subjective gain–loss contrast, whereas concern for a partner’s loss may partly offset the value of personal gains in competition. We therefore hypothesized that high-prosocial individuals would show a more negative dFRN in cooperation than in competition, reflecting stronger loss–gain differentiation. We did not make a strong directional prediction for low-prosocial individuals.

## Participants

We used G*Power software (Faul et al., 2009) to calculate the statistical power of the two-way analysis of variance (ANOVA; within-between interaction), with a preset *f* = 0.40 (η^2^ = 0.14, derived from a similar study by Wang et al., 2017). The analysis showed that when the sample size was 14 participants, the statistical power to detect interaction effects and main effects exceeded 90%. Ultimately, a total of 40 individuals (20 females and 20 males) were recruited for this study. All participants had normal or corrected-to-normal vision and no history of psychological disorders. The average age of the participants was 22 years (SD = 2.13). To minimize potential gender-related influences, each experimental task was conducted with pairs of participants of the same sex. All 40 participants took part in both the cooperative and competitive experiments. Participants received compensation proportional to the points they accumulated during the tasks. Because the sample was modest and sex was balanced overall but not within the prosociality groups, we report sex-controlled and continuous-score sensitivity analyses below.

**Figure 1:**
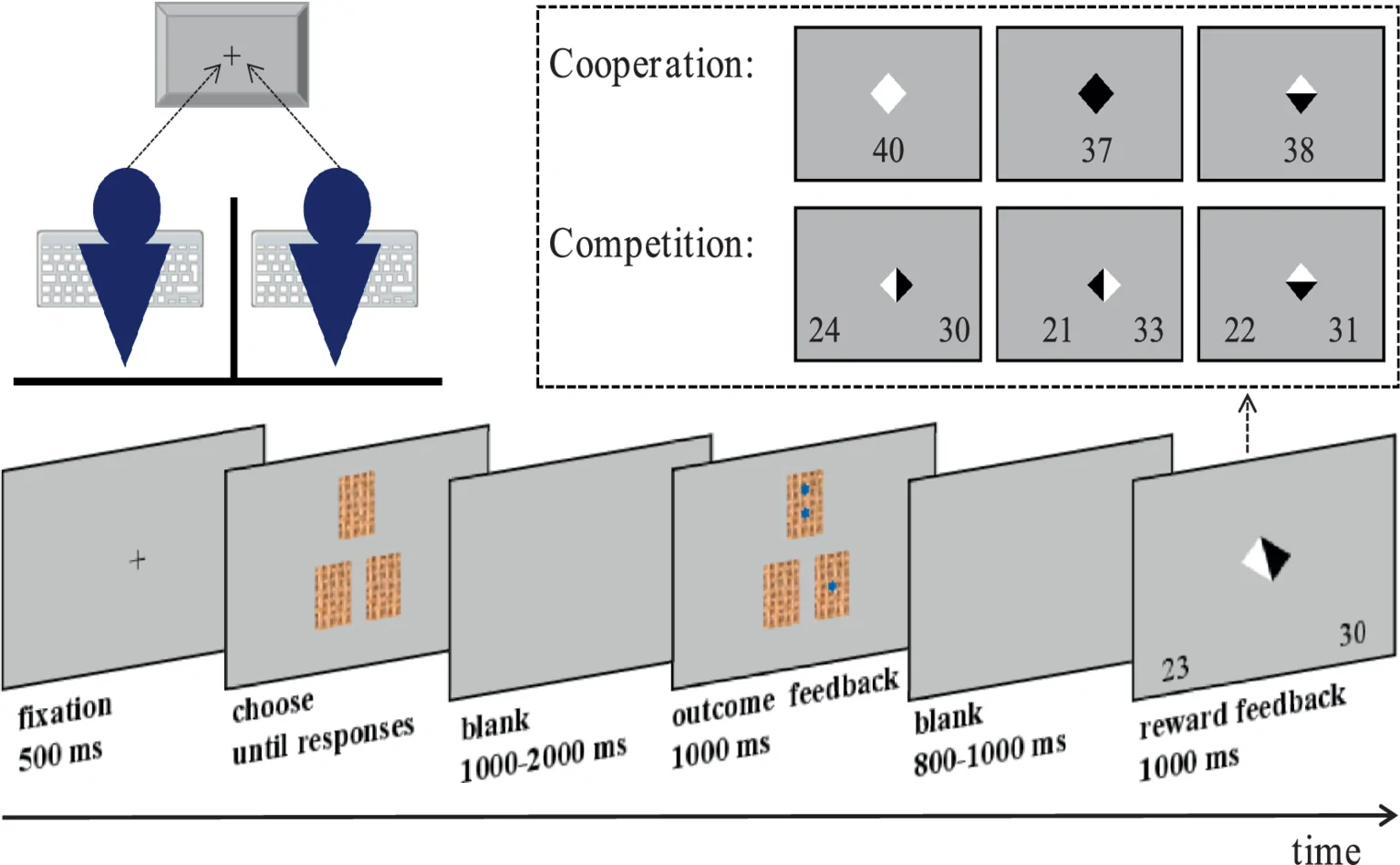
Experimental process. Two same-sex participants faced the same computer screen. They first completed the cooperation task and then carried out the competition task. In the cooperation task, both participants received the same outcome based on their joint card total: a total below 2 resulted in a loss of 1 point, a total of 2 resulted in neutral feedback, and a total above 2 resulted in a gain of 2 points. In the competition task, the participant selecting the card with more stars gained 2 points, the participant selecting the card with fewer stars lost 1 point, and equal selections produced neutral feedback. The cumulative scores of the participants were displayed in real time on the screen.

## Procedure

The experiment consists of two parts: a cooperative task and a competitive task, programmed using E-Prime software (Version 3.0). All instructions and stimuli were displayed on a 23-inch screen with a gray background, a resolution of 1,280 × 768 pixels, and a refresh rate of 60 Hz. Two participants were seated in front of the screen, separated by a partition. They could see each other but not each other’s hands or response buttons (which were covered with a towel), ensuring that neither would know the other’s choice.

Cooperative Task: Participants completed a task with shared outcome consequences. As shown in Figure 1, three cards were displayed on the screen, each hiding 0, 1, or 2 stars, and both participants simultaneously selected one card. The sum of the two selected cards determined the same feedback for both participants. If the total was below 2, each participant lost 1 point; if the total equaled 2, both received neutral feedback; and if the total exceeded 2, each participant gained 2 points. Thus, the task involved outcome-level interdependence because one participant’s feedback depended on both selections, although participants were not required to negotiate a strategy or form an explicit mutual goal. The procedure for a single trial was as follows: First, a fixation point was displayed for 500 ms, followed by the appearance of three cards (positioned at the top, left, and right sides of the screen). Participants selected a card using their respective keyboards. After both participants made their selections, following an inter-stimulus interval (ISI) of 1,000–2000 ms, the outcome feedback was displayed for 1,000 ms, revealing the number of stars on each card (participants could not see each other’s choices). Subsequently, after another ISI of 800–1,000 ms, the reward feedback was displayed for 1,000 ms. The cumulative score was displayed at the bottom of the screen, allowing participants to track their total earnings. At the beginning of the experiment, participants were informed that performance in the cooperative task would influence final payment, motivating them to engage seriously in the task. The interval between two consecutive trials was 1,000 ms. Before the formal experiment, participants completed 10 practice trials. The formal experiment consisted of 4 blocks, each containing 72 trials, with 1–3 min of rest between blocks to reduce fatigue.

Competitive Task: After completing the cooperative task, participants took a 5-min break before proceeding to the competitive task. The cumulative score from the cooperative task served as the starting score for the competitive task. The procedure was similar to that of the cooperative task, but the outcome was determined by the relative card values: the participant who selected the card with more stars gained 2 points, the participant who selected the card with fewer stars lost 1 point, and equal selections produced neutral feedback. The reward feedback indicated the participant who gained points, the participant who lost points, or a neutral outcome when the selections were equal. The cumulative score was displayed in the lower-left or lower-right corner of the screen, updating in real time. The competitive task also consisted of 4 blocks, each containing 72 trials, with 1–3 min of rest between blocks. Because cooperation and competition generated feedback using different operational rules, empirical feedback frequencies were not assumed to be equivalent across tasks.

All participants completed the cooperative task before the competitive task. This fixed order was chosen because performing the competitive task first might induce an antagonistic mindset toward the partner and thereby influence subsequent cooperation. Participants were not informed about the competitive task at the beginning of the experiment; it was introduced only after they had completed the cooperative task. Ultimately, compensation was determined based on the total scores in the competitive task. Although this sequence was intended to reduce the potential influence of prior competition on cooperation, it leaves order, fatigue, practice, and carryover effects inseparable from social context; this limitation is considered in the Discussion.

## The measurement of prosocial scores

The Prosocial Scale was employed to assess participants’ prosocial tendencies (Goodman et al., 1998). This scale consists of five items: (1) I usually share with others (e.g., games, food, pens, etc.); (2) I try to be kind to others; (3) I care about others’ feelings; (4) I am considerate toward younger children; and (5) I often volunteer to help others (e.g., parents, teachers, or peers). To avoid potential interference between the questionnaire and the experimental tasks (Job et al., 2013), participants completed the Prosocial Scale 1 week after the conclusion of the experiment. For each item, participants were asked to reflect on their experiences over the past 6 months and rate their responses on a scale ranging from “not true” (1 point) to “somewhat true” (2 points) to “certainly true” (3 points). Higher total scores indicate greater self-reported prosocial behavior (Li et al., 2021). For the primary categorical analysis, participants were divided at the sample median. Because median splitting discards continuous information, the original scores were also retained for a continuous-score sensitivity analysis.

## Electroencephalogram (EEG) recording and preprocessing

EEG data were acquired using a 64-electrode cap (Brain Products, Munich, Germany) arranged according to the international 10/20 system. The online reference electrode was positioned at FCz on the forehead, while the ground electrode was placed at AFz. A vertical electrooculogram (VEOG) was recorded using a facial electrode positioned approximately 1.5 cm below the right eye. All electrodes were amplified with an online band-pass filter set to 0.01–100 Hz, and data were continuously sampled at a rate of 1,000 Hz per channel. All electrode impedances were kept below 5 kΩ.

Offline preprocessing of the EEG data was conducted using the EEGLAB toolbox. The data were first re-referenced to the bilateral mastoid electrodes (TP9 and TP10). Subsequently, the signals were first processed with a 0.01–30 Hz band-pass filter (Cui et al., 2018). Independent Component Analysis (ICA) was applied to correct for major artifacts, including ocular movements, eye blinks, and muscle-related potentials (Chaumon et al., 2015; Flexer et al., 2005). The ICA decomposition was performed using the default runica algorithm in EEGLAB, which employs the extended Infomax ICA method (Dien et al., 2007). The ADJUST plug-in for EEGLAB was utilized to remove artifact-related ICA components based on the plug-in’s default criteria (Mognon et al., 2011). Additionally, ERP trials containing residual artifacts (defined as mean voltage values exceeding ±100 μV) were excluded from subsequent analyses.

Offline preprocessing of the EEG data was conducted using the EEGLAB toolbox. The data were first re-referenced to the bilateral mastoid electrodes (TP9 and TP10). Subsequently, the signals were first processed with a 0.01–30 Hz band-pass filter (Cui et al., 2018). Independent Component Analysis (ICA) was applied to correct for major artifacts, including ocular movements, eye blinks, and muscle-related potentials (Chaumon et al., 2015; Flexer et al., 2005). The ICA decomposition was performed using the default runica algorithm in EEGLAB, which employs the extended Infomax ICA method (Dien et al., 2007). The ADJUST plug-in for EEGLAB was utilized to remove artifact-related ICA components based on the plug-in’s default criteria (Mognon et al., 2011). Additionally, ERP trials containing residual artifacts (defined as mean voltage values exceeding ±100 μV) were excluded from subsequent analyses. Because each behavioral trial generated one reward-feedback ERP epoch, the behavioral records establish the trial denominator for the ERP analysis. Artifact-related rejection was below 5% overall, so more than 95% of recorded trials were retained. We therefore did not perform a separate inferential analysis of trial counts.

## Data processing and statistical analysis

Based on the grand average waveform and previous research (Lu et al., 2022), this study focused on the FRN component. Following difference-wave procedures (Huang et al., 2017; Gründler et al., 2009), dFRN was calculated as loss-evoked minus gain-evoked mean voltage, averaged across Fz, FCz, and Cz in the 220–300-ms interval after reward-feedback onset. More negative values therefore indicate stronger loss-minus-gain differentiation. The frontocentral midline sites were selected because they cover the characteristic distribution of feedback-related activity (Baker and Holroyd, 2011; Sambrook and Goslin, 2015). Neutral feedback did not enter this contrast. The sample median prosociality score was 11.5, with no ties at the cutoff. The low-prosocial group comprised scores of 6–11 (n = 20; M = 9.30, SD = 1.75; 9 females and 11 males), and the high-prosocial group comprised scores of 12–15 (n = 20; M = 13.70, SD = 1.17; 11 females and 9 males). A 2 (Social Context: cooperation and competition) × 2 (Prosociality: high and low) mixed ANOVA was conducted on dFRN. Partial eta squared (ηp^2^) was reported for ANOVA effects, and paired-sample Cohen’s dz. was calculated from the cooperation-minus-competition difference divided by the standard deviation of that difference. With only two within-participant levels, a sphericity correction was unnecessary.

To retain continuous information, a linear mixed-effects model used the two context-specific observations from each participant, with a participant random intercept. The sex-adjusted specification was dFRN ~ Context × Score + Context × Sex + (1 | Participant). Context was coded 1 for cooperation and 0 for competition, Score was centered at the sample mean of 11.5, and Sex was coded 1 for male and 0 for female. Thus, the Context × Score coefficient estimates how the cooperation-minus-competition contrast changes per one-point increase in prosociality. Models were fitted by restricted maximum likelihood, and two-sided Wald z tests and 95% confidence intervals were reported. As finite-sample sensitivity checks, participant difference scores were regressed on continuous score and sex, and separately on group and sex, using t tests with 37 residual degrees of freedom. The continuous difference-score regression was also evaluated using HC3 heteroscedasticity-robust standard errors. These reanalyses used Python 3.12, SciPy 1.18.1, and statsmodels 0.15.0. Statistical significance was defined as *p* < 0.05; 0.05 < *p* < 0.10 was described as marginal significance and interpreted as tentative evidence. No dyad random effect could be estimated because pair identifiers were unavailable in the analysis dataset.

**Figure 2:**
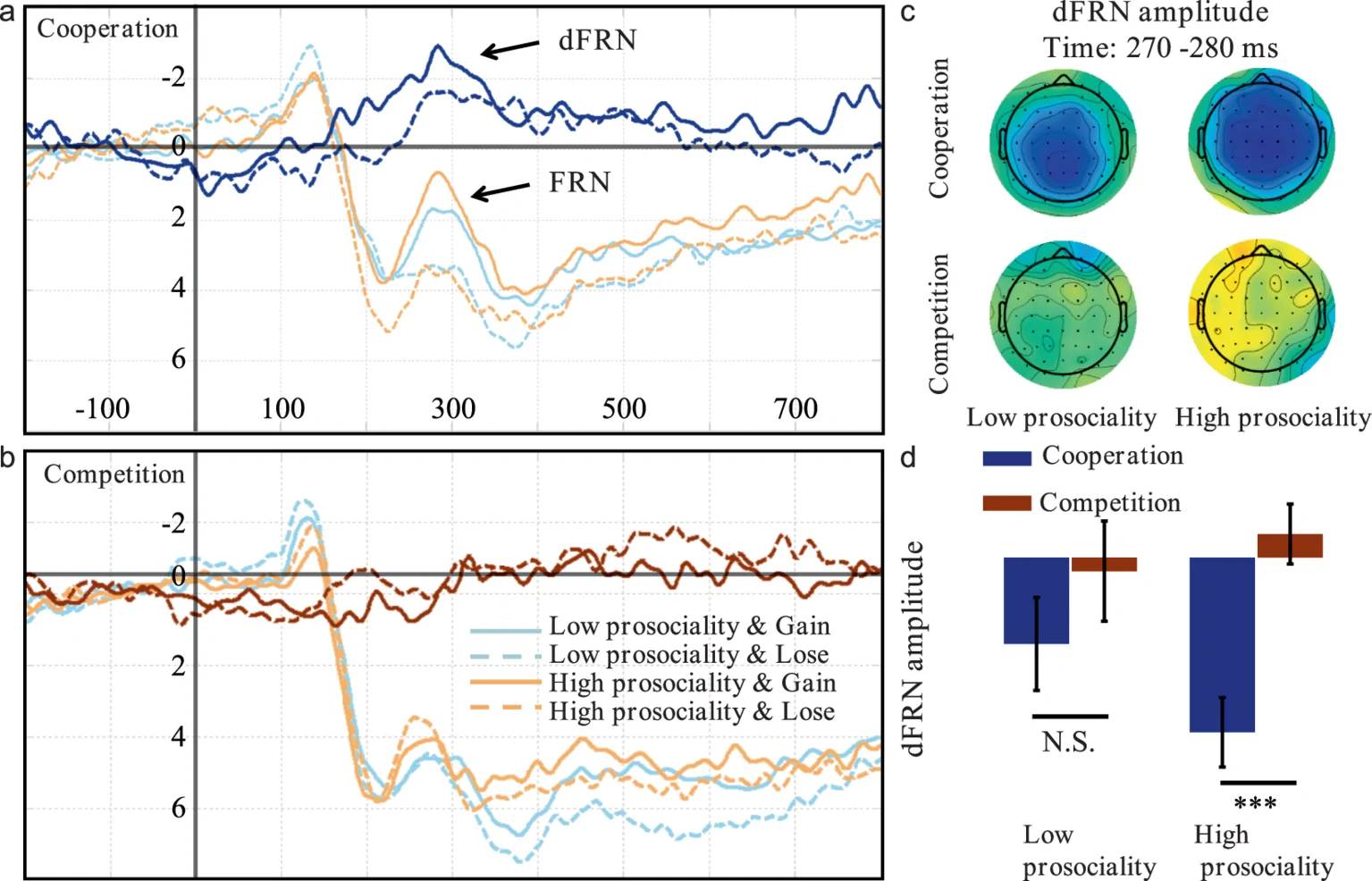
The results of event-related potential (ERP). **(a)** The average ERP waveforms evoked by reward outcomes in Fz, FCz, and Cz channels in the cooperative environment. **(b)** The average ERP waveforms evoked by reward outcomes in Fz, FCz, and Cz channels in the competitive environment. **(c)** The topographic map of the differential feedback-related negativity (dFRN). **(d)** The average dFRN amplitude within the 220–300-ms window in panels **(a,b)**. The black short bars represent the standard error. *** indicates *p* < 0.001.

## Results

### Behavioral results

Each context contained 288 trials per participant. Under the corrected cooperative rule, the nine possible joint card combinations were divided into three gain combinations—(1,2), (2,1), and (2,2)—three neutral combinations—(0,2), (1,1), and (2,0)—and three loss combinations—(0,0), (0,1), and (1,0). Thus, under independent and approximately equally likely card selections, each feedback category has a theoretical probability of 1/3, rather than cooperation producing only 32 gain trials. The competition rule also gives each participant a gain, loss, or neutral outcome in three of the nine relative-value combinations under the same assumption. The behavioral feedback records did not show a significant difference in the distribution of these outcome categories between contexts. The earlier statement that only (2,2) produced a gain was therefore a description error, not a feature of the task. Because the corrected rule does not imply the large gain/loss imbalance described in the review, and the behavioral check did not indicate a significant condition difference, we did not conduct an additional cooperation-specific frequency ANOVA. The two tasks nevertheless differed in how outcomes were generated and in their social consequences, so reward-frequency equivalence is not treated as evidence that the contexts were otherwise identical.

### ERP results

As shown in Figure 2, a two-way mixed ANOVA was conducted on mean dFRN amplitude averaged across Fz, FCz, and Cz in the 220–300-ms interval. In the low-prosocial group, the mean dFRN was −0.961 μV (SD = 2.687) in cooperation and −0.135 μV (SD = 1.979) in competition. In the high-prosocial group, the corresponding means were −2.126 μV (SD = 2.736) and 0.328 μV (SD = 1.770). The main effect of Social Context was significant, *F*(1, 38) = 17.17, *p* < 0.001, ηp^2^ = 0.311. The main effect of Prosocial Trait was not significant, *F*(1, 38) = 0.32, *p* = 0.576, ηp^2^ = 0.008. The Social Context × Prosocial Trait interaction was significant, *F*(1, 38) = 4.24, *p* = 0.046, ηp^2^ = 0.100. Follow-up paired comparisons showed a more negative dFRN in cooperation than in competition in the high-prosocial group, mean difference = −2.455 μV, 95% CI [−3.768, −1.142], t(19) = −3.91, *p* < 0.001, dz. = −0.875. The low-prosocial group’s context difference was not significant, mean difference = −0.826 μV, 95% CI [−1.837, 0.186], t(19) = −1.71, *p* = 0.104, dz. = −0.382. The negative signs follow the cooperation-minus-competition contrast.

In the continuous-score mixed-effects model without sex terms, the Context × Score coefficient was b = −0.320 μV per scale point, SE = 0.150, z = −2.14, *p* = 0.032. Including both Sex and Context × Sex yielded b = −0.315, SE = 0.152, z = −2.07, *p* = 0.039, 95% CI [−0.614, −0.016]. The Context × Sex term was not significant (*p* = 0.778). These estimates indicate that higher prosociality scores were associated with a more negative cooperation-minus-competition dFRN contrast. The finite-sample regression on participant difference scores yielded the same adjusted score coefficient, t(37) = −2.07, *p* = 0.046, 95% CI [−0.624, −0.006]. Using HC3 robust standard errors, this result was marginally significant, SE = 0.156, t(37) = −2.02, *p* = 0.051, 95% CI [−0.631, 0.001]. In the group-based difference-score regression adjusted for sex, the group contrast was also marginally significant, b = −1.604 μV, SE = 0.805, t(37) = −1.99, *p* = 0.054, 95% CI [−3.236, 0.028]; the sex coefficient in this model was not significant (*p* = 0.753). The consistent direction across specifications supports a context-dependent association, but the strength of evidence is sensitive to the statistical specification.

## Discussion

This study examined how prosociality relates to reward processing during cooperative and competitive tasks in a face-to-face interaction paradigm. The primary analysis showed a Social Context × Prosocial Trait interaction: high-prosocial individuals had a more negative dFRN in cooperation than in competition, whereas the low-prosocial group did not show a statistically reliable context difference. The continuous-score mixed-effects analysis yielded a comparable association after adjustment for sex. The categorical sex-adjusted analysis and the continuous analysis with robust standard errors were marginally significant. Together, these findings are consistent with context-dependent reward differentiation associated with prosociality, while the strength of evidence warrants caution. The fixed task order, differing outcome-generation rules, and unmodeled dependence between partners limit causal interpretation.

The observed context-by-prosociality pattern is consistent with the proposed difference in the subjective value of social outcomes. Shared gains and losses have aligned implications for the two partners, whereas a personal gain in competition accompanies the partner’s loss. Greater concern for the partner could consequently increase the contrast between shared gains and shared losses relative to competitive outcomes. Expectancy-based accounts offer a possible additional explanation: a shared loss may be more discrepant if successful joint outcomes were expected (Holroyd and Coles, 2002; Pfabigan et al., 2011). However, expectations were not measured, and a preference for cooperation cannot be equated with a higher perceived probability of cooperative success. Because dFRN contrasts two responses, the present effect also cannot reveal whether expectations selectively changed loss responses, gain responses, or both.

Motivational weighting provides a complementary interpretation. The association between subjective involvement and feedback negativity reported by Yeung et al. (2005) supports the possibility that personally relevant outcomes receive stronger evaluative processing. For high-prosocial individuals, joint outcomes may carry greater motivational significance, producing stronger loss–gain differentiation during cooperation (Godman et al., 2014). A more negative dFRN could also reflect a larger reward positivity to shared gains (Proudfit, 2015), rather than an exclusively enhanced response to losses. These possibilities are consistent with the value-based account but cannot be distinguished using the difference score alone. The study did not directly measure subjective value, happiness, or sadness, and the competition pattern does not establish an inverse reward response.

Existing studies have reported inconsistent effects of cooperative tasks on reward sensitivity. Traditional paradigms (Kimura and Katayama, 2016; Lei et al., 2019) often use computer-simulated cooperation and report weaker FRN responses, whereas real face-to-face paradigms may produce stronger engagement (Yokota and Naruse, 2022). The present findings are consistent with the possibility that physical co-presence and shared consequences increase the salience of cooperative feedback for high-prosocial individuals. Nevertheless, because the cooperative and competitive tasks were completed in a fixed order and did not use identical outcome-generation rules, the present study cannot isolate the effect of social context from order, fatigue, carryover, or feedback-frequency differences. These methodological features qualify the interpretation of the context effect.

Competitive environments also involve social comparison (Kang et al., 2013; Ruff and Fehr, 2014; Gerber et al., 2018; Luo et al., 2018), which can shift outcome evaluation from absolute gains to relative outcomes. In the present study, the preceding cooperation task may have changed the interpersonal relationship before competition began, potentially affecting later comparison processes (Lubbers et al., 2009; Sun et al., 2023). The data do not provide sufficient evidence to conclude that competitive success was experienced negatively, that high-prosocial individuals showed an inverse reward response, or that competition generally reduced reward sensitivity. The observed competition-related pattern is better described as a context-dependent change in loss-minus-gain differentiation. Direct measures of social comparison, subjective value, and affect would be needed to distinguish these explanations.

This study has several limitations. First, cooperation always preceded competition to reduce the risk that competition-induced antagonism toward the partner would influence subsequent cooperation. Social context is consequently confounded with task order. Rest periods cannot eliminate fatigue, practice, or carryover effects, and an interaction with prosociality does not rule out differential susceptibility to these effects. Carrying the cooperative score into competition may also have affected expectations and outcome value. Future studies should counterbalance task order or use separate groups and independent score baselines. Second, both tasks used gains of 2 points, losses of 1 point, and neutral feedback, but cooperation depended on a joint total whereas competition depended on relative card values. The social consequences and feedback displays also differed. Without observed feedback frequencies and retained ERP trial counts, reward-frequency and signal-reliability explanations cannot be excluded. Third, participants completed the task in pairs, but pair identifiers were unavailable for this reanalysis. The participant random intercept accounts for repeated observations within an individual; it does not account for dependence between partners. The precision of the present estimates may therefore be overstated if dyad-level dependence is substantial.

The median split distinguished relatively lower and higher scores within this sample rather than two discrete types of prosociality. Dichotomization discards information, and the five-item, three-point self-report measure offers limited score resolution. Continuous-score modeling retained this information and yielded an association in the predicted direction after adjustment for sex. However, the categorical sex-adjusted analysis (*p* = 0.054) and the HC3 robust continuous analysis (*p* = 0.051) were marginally significant. The sample was modest, and the planning calculation based on a large assumed effect does not establish adequate power for smaller interactions or precise effect estimation. Item-level questionnaire responses were not available for a sample-specific reliability estimate. The absence of a significant context difference in the low-prosocial group should not be interpreted as evidence that the effect is absent. Larger studies using continuous scores, more comprehensive trait measures, and identifiable dyads are needed to assess the stability and generalizability of the findings.

Sex may also influence outcome evaluation in social contexts. Including Sex and Context × Sex in the continuous mixed-effects model retained the prosociality-by-context association (*p* = 0.039), while the categorical sex-adjusted contrast was marginally significant (*p* = 0.054) (Espinosa and Kovarik, 2015; Kamas and Preston, 2021). The non-significant Context × Sex term does not establish an absence of sex differences. Balanced samples with sufficient power to examine these interactions are needed. More broadly, the present results are consistent with prosociality-related differences in the motivational weighting of cooperative outcomes, but expectancy, subjective affect, and social value orientation were not directly measured. These remain possible explanations rather than demonstrated mechanisms.

## Data Availability

The datasets presented in this study can be found in online repositories. The names of the repository/repositories and accession number(s) can be found in the article/supplementary material.
